# Supplementary material for: Unveiling the Bioactive Potential of the Invasive Jellyfish Phyllorhiza punctata Through Integrative Transcriptomic and Proteomic Analyses
Source: Biomolecules. 2025 Aug 4;15(8):1121. doi: 10.3390/biom15081121 (PMC12383608; doi:10.3390/biom15081121)
Supplement: Supplementary file 1 [file biomolecules-15-01121-s001.zip › Suplementary Material/FileS3/PEPFOLD4/XP_065060115_8203/clusters.html]

 PEP-FOLD clusters


Download archive of all models  here   

|  |  |  |
| --- | --- | --- |
| # | file | sOPEP |
| # cluster 1 - Size: 30 |  |  |
|  | XP\_065060115\_8203-model1 | -17.4772 |
|  | XP\_065060115\_8203-model1.1 | -17.4772 |
|  | XP\_065060115\_8203-model1.2 | -17.4373 |
|  | XP\_065060115\_8203-model1.3 | -17.4373 |
|  | XP\_065060115\_8203-model1.4 | -17.4373 |
|  | XP\_065060115\_8203-model1.5 | -17.4373 |
|  | XP\_065060115\_8203-model1.6 | -17.4373 |
|  | XP\_065060115\_8203-model1.7 | -17.4373 |
|  | XP\_065060115\_8203-model1.8 | -17.4373 |
|  | XP\_065060115\_8203-model1.9 | -17.4225 |
|  | XP\_065060115\_8203-model1.10 | -17.4225 |
|  | XP\_065060115\_8203-model1.11 | -17.4225 |
|  | XP\_065060115\_8203-model1.12 | -17.4225 |
|  | XP\_065060115\_8203-model1.13 | -17.4041 |
|  | XP\_065060115\_8203-model1.14 | -17.4041 |
|  | XP\_065060115\_8203-model1.15 | -17.4041 |
|  | XP\_065060115\_8203-model1.16 | -17.3893 |
|  | XP\_065060115\_8203-model1.17 | -17.3893 |
|  | XP\_065060115\_8203-model1.18 | -17.3893 |
|  | XP\_065060115\_8203-model1.19 | -17.3862 |
|  | XP\_065060115\_8203-model1.20 | -17.3387 |
|  | XP\_065060115\_8203-model1.21 | -17.3387 |
|  | XP\_065060115\_8203-model1.22 | -17.2228 |
|  | XP\_065060115\_8203-model1.23 | -17.2196 |
|  | XP\_065060115\_8203-model1.24 | -17.2101 |
|  | XP\_065060115\_8203-model1.25 | -17.205 |
|  | XP\_065060115\_8203-model1.26 | -17.1812 |
|  | XP\_065060115\_8203-model1.27 | -17.1498 |
|  | XP\_065060115\_8203-model1.28 | -17.131 |
|  | XP\_065060115\_8203-model1.29 | -17.0694 |
| # cluster 2 - Size: 1 |  |  |
|  | XP\_065060115\_8203-model2 | -9.54638 |
| # cluster 3 - Size: 3 |  |  |
|  | XP\_065060115\_8203-model3 | -9.2921 |
|  | XP\_065060115\_8203-model3.1 | -9.06069 |
|  | XP\_065060115\_8203-model3.2 | -8.28854 |
| # cluster 4 - Size: 9 |  |  |
|  | XP\_065060115\_8203-model4 | -8.85282 |
|  | XP\_065060115\_8203-model4.1 | -8.74363 |
|  | XP\_065060115\_8203-model4.2 | -8.73015 |
|  | XP\_065060115\_8203-model4.3 | -8.71322 |
|  | XP\_065060115\_8203-model4.4 | -8.70531 |
|  | XP\_065060115\_8203-model4.5 | -8.52401 |
|  | XP\_065060115\_8203-model4.6 | -8.20837 |
|  | XP\_065060115\_8203-model4.7 | -8.20837 |
|  | XP\_065060115\_8203-model4.8 | -6.84267 |
| # cluster 5 - Size: 2 |  |  |
|  | XP\_065060115\_8203-model5 | -8.83732 |
|  | XP\_065060115\_8203-model5.1 | -8.81584 |
| # cluster 6 - Size: 3 |  |  |
|  | XP\_065060115\_8203-model6 | -6.09676 |
|  | XP\_065060115\_8203-model6.1 | -5.69633 |
|  | XP\_065060115\_8203-model6.2 | -5.52059 |
| # cluster 7 - Size: 4 |  |  |
|  | XP\_065060115\_8203-model7 | -5.94115 |
|  | XP\_065060115\_8203-model7.1 | -5.67996 |
|  | XP\_065060115\_8203-model7.2 | -5.6219 |
|  | XP\_065060115\_8203-model7.3 | -5.32424 |
| # cluster 8 - Size: 13 |  |  |
|  | XP\_065060115\_8203-model8 | -5.93246 |
|  | XP\_065060115\_8203-model8.1 | -5.8013 |
|  | XP\_065060115\_8203-model8.2 | -5.74248 |
|  | XP\_065060115\_8203-model8.3 | -5.7127 |
|  | XP\_065060115\_8203-model8.4 | -5.55986 |
|  | XP\_065060115\_8203-model8.5 | -5.53961 |
|  | XP\_065060115\_8203-model8.6 | -5.53785 |
|  | XP\_065060115\_8203-model8.7 | -5.48467 |
|  | XP\_065060115\_8203-model8.8 | -5.43124 |
|  | XP\_065060115\_8203-model8.9 | -5.36954 |
|  | XP\_065060115\_8203-model8.10 | -5.29307 |
|  | XP\_065060115\_8203-model8.11 | -5.26417 |
|  | XP\_065060115\_8203-model8.12 | -5.19971 |
| # cluster 9 - Size: 5 |  |  |
|  | XP\_065060115\_8203-model9 | -5.8431 |
|  | XP\_065060115\_8203-model9.1 | -5.77926 |
|  | XP\_065060115\_8203-model9.2 | -5.56541 |
|  | XP\_065060115\_8203-model9.3 | -5.55833 |
|  | XP\_065060115\_8203-model9.4 | -5.37962 |
| # cluster 10 - Size: 3 |  |  |
|  | XP\_065060115\_8203-model10 | -5.76325 |
|  | XP\_065060115\_8203-model10.1 | -5.67667 |
|  | XP\_065060115\_8203-model10.2 | -5.234 |
| # cluster 11 - Size: 5 |  |  |
|  | XP\_065060115\_8203-model11 | -5.71259 |
|  | XP\_065060115\_8203-model11.1 | -5.66328 |
|  | XP\_065060115\_8203-model11.2 | -5.64548 |
|  | XP\_065060115\_8203-model11.3 | -5.43944 |
|  | XP\_065060115\_8203-model11.4 | -5.43525 |
| # cluster 12 - Size: 7 |  |  |
|  | XP\_065060115\_8203-model12 | -5.64467 |
|  | XP\_065060115\_8203-model12.1 | -5.55059 |
|  | XP\_065060115\_8203-model12.2 | -5.47189 |
|  | XP\_065060115\_8203-model12.3 | -5.39545 |
|  | XP\_065060115\_8203-model12.4 | -5.32942 |
|  | XP\_065060115\_8203-model12.5 | -5.32052 |
|  | XP\_065060115\_8203-model12.6 | -5.25317 |
| # cluster 13 - Size: 6 |  |  |
|  | XP\_065060115\_8203-model13 | -5.50852 |
|  | XP\_065060115\_8203-model13.1 | -5.35691 |
|  | XP\_065060115\_8203-model13.2 | -5.2415 |
|  | XP\_065060115\_8203-model13.3 | -5.05343 |
|  | XP\_065060115\_8203-model13.4 | -5.04336 |
|  | XP\_065060115\_8203-model13.5 | -4.97179 |
| # cluster 14 - Size: 6 |  |  |
|  | XP\_065060115\_8203-model14 | -5.42951 |
|  | XP\_065060115\_8203-model14.1 | -5.3122 |
|  | XP\_065060115\_8203-model14.2 | -5.22422 |
|  | XP\_065060115\_8203-model14.3 | -5.1718 |
|  | XP\_065060115\_8203-model14.4 | -5.14074 |
|  | XP\_065060115\_8203-model14.5 | -5.09807 |
| # cluster 15 - Size: 1 |  |  |
|  | XP\_065060115\_8203-model15 | -5.2446 |
| # cluster 16 - Size: 1 |  |  |
|  | XP\_065060115\_8203-model16 | -5.22802 |
| # cluster 17 - Size: 1 |  |  |
|  | XP\_065060115\_8203-model17 | -5.16931 |
|  |  |  |
